# Variation in trophic niches of oribatid mites in temperate forest ecosystems as indicated by neutral lipid fatty acid patterns

**DOI:** 10.1007/s10493-020-00494-2

**Published:** 2020-04-28

**Authors:** Mark Maraun, Dana Augustin, Melanie M. Pollierer, Stefan Scheu

**Affiliations:** 1 J.F. Blumenbach Institute of Zoology and Anthropology, University of Göttingen, Untere Karspüle 2, 37073 Göttingen, Germany; 2 Centre of Biodiversity and Sustainable Land Use (CBL), University of Göttingen, Von-Siebold-Str. 8, 37075 Göttingen, Germany

**Keywords:** Trophic niche, Global change, Oribatida, NLFA

## Abstract

Shifting of trophic niches of soil microarthropods may allow them to adapt to changing resource conditions as induced by global change processes. However, the capability of microarthropods to shift their trophic niches is little studied. Whereas some studies based on stable isotopes (^15^N/^13^C) point to distinct and narrow trophic niches, others indicate that trophic niches are plastic. Here, we investigated shifts in trophic niches of oribatid mites (Oribatida, Acari), a major soil detritivore microarthropod group, due to forest management, i.e., plantation of beech and spruce forests in Central Europe, using neutral lipid fatty acid (NLFA) markers. Due to differential microbial communities, we expected a fungi-based diet of oribatid mites in beech forest, but more bacterial contributions to the diet in spruce forest. Supporting these hypotheses, NLFA markers indicated that the trophic niches of each of the studied oribatid mite species differed between beech and spruce forests and shifted from feeding predominantly on litter and fungi in beech forest to more intensively feeding on bacteria in spruce forest. Oribatid mite species with the most pronounced differences in trophic niches included *Achipteria coleoptrata*, *Eupelops hirtus*, *Eupelops plicatus* and *Liacarus xylariae*, which had been classified as primary or secondary decomposers in previous studies. Overall, the results indicate that the ability of oribatid mite species to colonize different habitats and ecosystems is due to their ability to adjust their diet, i.e., to trophic plasticity. Changes in trophic niches in each of the studied oribatid mite species suggest that detritivores in soil may better cope with future changes in environmental conditions and associated changes in resource composition than species above the ground.

**Electronic supplementary material:**

The online version of this article (10.1007/s10493-020-00494-2) contains supplementary material, which is available to authorized users.

## Introduction

Shifting of trophic niches allows animals to cope with fluctuating or changing resource conditions. It has been assumed to be especially important in habitats with either low quality or low amounts of resources (MacNeil et al. 1997). In addition, shifting trophic niches may allow species to cope with changing abiotic conditions as these may indirectly affect resource availability and accessibility (Carreira et al. 2016). Trophic plasticity therefore is an important trait allowing species to cope with global change processes such as increasing temperature or increasing frequency of extreme climatic events (Hoffmann and Sgrò 2011). Some animal species may evade changing resource conditions by moving to other habitats, but many species are limited by dispersal (Riibak et al. 2015), or may be inferior competitors and not survive in new habitats (Hågvar et al. 1990). Additionally, new habitats may not be easily available in the first place (Meyer et al. 2015). Consequently, the degree of trophic plasticity in animal taxa is likely to be a major determinant of their survival in a changing world.

Until today, only few studies investigated the ability of animal taxa to respond to changing resource conditions by shifting their trophic niches. For instance, the intertidal gastropod *Peringia* (*Hydrobia*) *ulvae* consumes allochthonous detritus and bacteria in sandy and muddy sediments, but switches to autochthonous detritus from *Spartina maritima* in salt marsh habitats (Riera 2019). Furthermore, larvae of Mediterranean amphibians change their diet in the presence of competitors when the preferred food (macrophytes) is reduced in density and instead consume detritus, algae and zooplankton (Arribas et al. 2015). Furthermore, resource use of colonies of ant species in a tropical rainforest in Australia has been shown to vary in space (Blüthgen et al. 2003). However, studies investigating the ability of animals of the temperate zone to shift trophic niches are rare; especially trophic plasticity in soil animals is poorly studied, which is surprising considering their importance for decomposition processes and thus for ecosystem functioning. The ability to adapt to changing local environmental conditions may be particularly important for soil animals as they typically are less mobile than species above the ground and therefore their ability to adapt to changes, e.g. in temperature, by changing their distributional range is likely to be limited (Lehmitz et al. 2011, 2012).

Temperate forests in Europe have undergone intense range shifts during the last ice ages (Bolte et al. 2007), but also in more recent periods such as the Little Ice Age in the sixteenth to nineteenth century (Fagan 2000). Animals living in these forests likely have experienced severe changes in abiotic conditions as well as resource availability (Hågvar et al. 2009), and therefore may respond in a flexible way to harsh and changing abiotic conditions and changes in resource supply. In fact, predatory beetles of temperate forests, such as Carabidae, have a wide trophic range as indicated by variations in stable isotope ratios (Zalewski et al. 2016). However, the degree of trophic plasticity has hardly been investigated in animals of temperate forests, especially in those living in soil.

Soil animals arguably are the most important components of the animal community in temperate forest ecosystems (Lavelle et al. 2006). Together with microorganisms, they recycle plant residues and thereby significantly contribute to ecosystem functioning (Peterson and Luxton 1982; Schaefer 1991). Oribatid mites are among the most abundant and diverse soil animal taxa (Maraun and Scheu 2000; Maraun et al. 2007), worldwide more than 10,000 species have been described (Schatz 2002; Subias 2004, 2016; Schatz and Behan-Pelletier 2007). They feed on a wide variety of resources, ranging from dead organic material to lichens, algae, bacteria and fungi to nematodes (Maraun et al. 2011). Their trophic ecology has been studied in the last decades using a variety of methods including stable isotope, fatty acid and molecular gut content analyses (Schneider et al. 2004; Heidemann et al. 2011; Pollierer et al. 2012; Gong et al. 2018). Fischer et al. (2014) pointed out that the high variation in stable isotope signatures, as found in oribatid mites, points to trophic plasticity. However, trophic plasticity in oribatid mites has been little studied and existing studies yielded contradictory results. Using stable isotopes (^15^N/^13^C) Gan et al. (2014) found trophic plasticity to be limited, whereas Coral-Hernandez et al. (2015) and Krause et al. (2019) concluded that trophic plasticity in oribatid mite species is high. These contradictory findings underline the necessity for further investigations allowing more detailed insight into the variability of trophic niches.

Fatty acids, especially neutral lipid fatty acids (NLFAs), have been used as biomarkers to investigate the nutrition of and trophic relationships between soil animals such as collembolans and oribatid mites (Ruess et al. 2004; van Dooremalen and Ellers 2010; Ruess and Chamberlain 2010; Pollierer et al. 2010; Ferlian et al. 2015; Brückner et al. 2017; Kühn et al. 2019). Typically, fatty acids are incorporated from the diet directly into storage lipids of consumers without modification (‘dietary routing’; Blem 1976), thereby providing time-integrated information on animal diets (Haubert et al. 2011). Marker fatty acids can be used to separate the relative contribution of the fungal, bacterial and plant energy channel to soil animal nutrition (Ruess and Chamberlain 2010), thereby complementing stable isotope analyses by allowing to trace basal resources (Traugott et al. 2013; Potapov et al. 2019). For instance, Pollierer et al. (2012) inferred the relative importance of trophic channels for soil animals using the relative abundance of NLFA biomarkers and showed that the importance of the bacterial channel in forest soil animal food webs has been underestimated. Compared to molecular gut content analysis, lipid analysis provides the advantage to reflect assimilated food resources, whereas the former reflects ingested materials irrespective of its assimilation. In addition, molecular gut content analysis depends on selected primers and comparisons across multiple taxa are not straight-forward (Traugott et al. 2013).

Few studies investigated trophic plasticity in animals by analyzing fatty acid profiles. Based on lipid analysis the coral *Stylophora subseriata* has been assumed to respond in a plastic way to changes in food sources due to global change events (Seemann et al. 2013). However, lipids rarely have been used for investigating variations in trophic niches of soil animals (Ferlian and Scheu 2014; Ferlian et al. 2015). In this study, we investigated shifts in trophic niches of soil living oribatid mite species with forest types, i.e., replacement of beech by spruce forests in Central Europe. The aim of our study was to investigate if soil-living oribatid mites adapt their trophic niches, allowing them to colonize different forest ecosystems and to cope with future environmental changes. Generally, we hypothesized that (1) the relative importance of the fungal, bacterial and plant energy channel for oribatid mite nutrition (as indicated by NLFAs) varies between sites with different tree species (beech and spruce). More specifically, we hypothesized that (2) oribatid mites in the studied beech forest feed more on fungi, whereas in the studied spruce forest they feed more on bacteria, thereby following the relative abundance of fungi and bacteria in these forests (Pollierer et al. 2015). Since nitrogen concentrations, and therefore the quality of beech litter, exceeds that of spruce litter (Zeller et al. 2007), we further hypothesized that (3) the relative importance of the plant channel for oribatid mite nutrition in the studied beech forest exceeds that in the studied spruce forest.

## Materials and methods

### Study site

Samples were taken in the Hainich forest in the framework of the Biodiversity Exploratories, a long-term research project investigating biodiversity – ecosystem functioning relationships (www.biodiversity-exploraties.de). The Hainich is a hilly region in central Germany (285–550 m a.s.l.); parent rock is mainly Triassic limestone. Luvisol is the main soil type; Cambisols and Stagnosols also occur. Soil pH averages 4.59 ± 0.67, annual precipitation is 500–800 mm and the mean annual temperature is 6.5–8.0 °C (Fischer et al. 2010a, b).

### Sampling

Twelve soil samples were taken from a ca. 70 year old coniferous forest (spruce, *Picea abies*) and a 70 year old deciduous forest (beech, *Fagus sylvatica*) in the Hainich region, Central Germany, in April 2014. The two forests were spaced by ca. 5 km. In each forest, samples were taken from an area of 1 m^2^, separated into litter and soil (upper 5 cm) and transferred into the laboratory. Samples within each forest were spaced by at least 300 m. Soil animals were extracted by heat (Kempson et al. 1963) and stored in 70% ethanol until determination using Weigmann (2006). For fatty acid (FA) analysis oribatid mite species from litter and soil were pooled as, based on results of stable isotope studies, trophic niches of oribatid mite species differ little between litter and soil (Scheu and Falca 2000). Only species with body size > 300 µm were used for the analyses. They included eight species that occurred in both forest types [*Achipteria coleoptrata* (11 samples from beech, 12 from spruce), *Damaeus riparius* (7, 6), *Euzetes globulus* (1, 3), *Eupelops hirtus* (6, 11), *Eupelops plicatus* (7, 3), *Liacarus xylariae* (2, 2), *Nothrus palustris* (4, 1) and *Platynothrus peltifer* (4, 5)], five which only occurred in beech forests [*Hermannia gibba* (2 samples), Phthiracaridae (7), *Steganacarus magnus* (8), *Tritegeus bisulcatus* (3), *Xenillus tegeocranus* (2)], and three that only occurred in spruce forests [*Adoristes ovatus* (3), *Edwardzetes edwardzi* (7), *Liacarus coracinus* (2); for details see Online Appendix 1].

### Fatty acid analysis

Depending on the size of the species and on the availability in each sample, 20–50 individuals were pooled for FA extraction. The number of individuals pooled was based on preliminary studies on the amount of tissue needed for FA analyses (D. Augustin, unpubl. data). Neutral lipid fatty acids (NLFAs) were extracted as described in Haubert et al. (2004). After extraction, neutral lipids were dried at 50 °C using a rotation vacuum concentrator (RVC 2e25, Christ, Osterode am Harz, Germany). Then, lipids were saponified, methylated and washed according to the protocol of the Sherlock Microbial Identification System (MIDI, Newark, NJ, USA). Obtained FA methyl esters were transferred into vials, capped and stored at − 21 °C until analysis via gas chromatography. The gas chromatograph (Clarus 500, Perkin Elmer, Waltham, MA, USA) was equipped with a flame ionisation detector (FID) and a PE-5 capillary column (30 m × 0.32 mm i.d., 0.25 mm film thickness, Perkin Elmer). Helium was used as carrier gas. More details on the analysis program are given in Ferlian and Scheu (2014). FA methyl esters (FAMEs) were identified by comparing retention times of samples with those of two standard mixtures, the first comprising 37 different FAMEs (Supelco 37 Component FAME Mix, Sigma Aldrich, St Louis, MO, USA) and the second 26 bacterial acid methyl esters (BAME Mix; Sigma Aldrich). The following FAs served as biomarkers for bacteria: the methyl-branched FAs i15:0 and a15:0, the cyclic FAs cy17:0 and cy19:0, and the unsaturated FAs 16:1ω7 and 18:1ω7 (Ruess and Chamberlain 2010). The unsaturated FAs 18:1ω9 and 18:2ω6,9 served as relative markers for plants and fungi, respectively, with a high proportion of 18:2ω6,9 indicating a fungal-biased diet, and a high relative proportion of 18:1ω9 indicating a plant-biased diet (Ruess et al. 2004; Ngosong et al. 2009; Pollierer et al. 2012; Kühn et al. 2019). The polyunsaturated fatty acid (PUFA) 18:3ω6,9,12 was used as marker for plants and algae (Kharlamenko et al. 1995; Ruess et al. 2007), and the PUFA 20:4ω6,9,12,15 as marker for algae (Kharlamenko et al. 1995) as well as animals and protists (Ruess and Chamberlain 2010; Comeault et al. 2010). The unsaturated fatty acid 22:1ω9 occurs in plants but also in animal species (Salvo et al. 2018). Furthermore, the non-specific FAs 14:0, 16:0, 17:0 and 18:0 were included in analyzing dietary niches of species. Fatty acids that made up less than 0.1% of the total fatty acid content (mean of all single measurements) were excluded from the analysis. After exclusion of these rare fatty acids, all fatty acid data were normalized to 100% (Online Appendix; Table 1). Although given as percentages, we did not transform fatty acid data as arcsin square-root transformations can produce “nonsensical predicted values” (Warton and Hui 2011), and logit transformation downweights abundant and important FAs, such as 18:2ω6,9 and 18:1ω9 with relative abundances typically in the range of 20–50%, and upgrades rare ones, which distorts the data.

### Statistical analyses

The fatty acid composition of all studied species from the beech and spruce forests was analysed by Non-Metric Multidimensional Scaling (NMDS) (R Development Core Team 2018) with Bray–Curtis as distance matrix. Therefore, the mean relative concentration of fatty acids of the 11 species/taxa from spruce forests (*Achipteria coleoptrata*, *Adoristes ovatus*, *Damaeus riparius*, *Edwardzetes edwardsi*, *Eupelops hirtus*, *Eupelops plicatus*, *Euzetes globulus*, *Liacarus coracinus*, *Liacarus xylariae*, *Nothrus palustris*, *Platynothrus peltifer*) and the 13 species/taxa from beech forests (*Achipteria coleoptrata*, *Damaeus riparius*, *Eupelops hirtus*, *Eupelops plicatus*, *Euzetes globulus*, *Hermannia gibba*, *Liacarus xylariae*, *Nothrus palustris*, Phthiracaridae, *Platynothrus peltifer*, *Steganacarus magnus*, *Tritegeus bisulcatus*, *Xenillus tegeocranus*) were calculated. The values of the first four NMDS axes then were used as dependent variables in Discriminant Function Analysis (DFA) to compare the fatty acid composition of species from beech and spruce forests. Forest type was used as grouping variable. The DFA was carried out in Statistica 13.3 (TIBCO Data Science).

Further, the relative abundance of FAs in oribatid mite species was analysed by Principal Components Analysis (PCA) as implemented in CANOCO 5.02 (Microcomputer Power, Ithaca, NY, USA, 2012). Oribatid mite species of the two forests were included as passive variables. In addition, the FA composition of six species that occurred in at least two samples per forest type (*Achipteria coleoptrata*, *Damaeus riparius*, *Eupelops hirtus*, *Eupelops plicatus*, *Platynothrus peltifer*, *Liacarus xylariae*) was analysed by NMDS. The first four NMDS axes were used for subsequent DFA for inspecting differences between beech and spruce forests (see above). In *Liacarus xylariae* the number of dimensions in the NMDS was reduced to two due to the small number of replicates.

## Results

### Fatty acid composition of oribatid mites

**Fig. 1:**
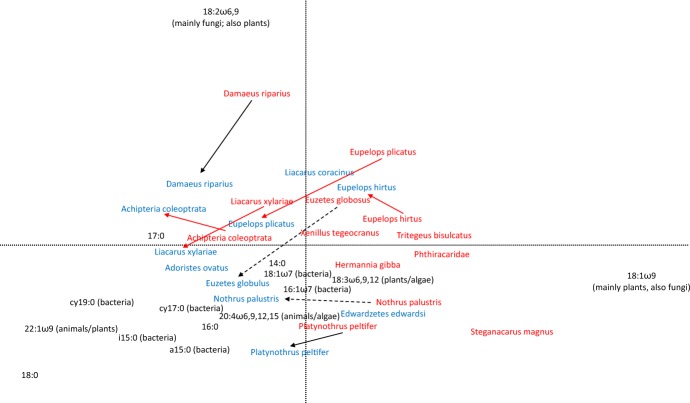
Principal component analysis of oribatid mite species from beech forest (red) and spruce forest (blue) based on their fatty acid (NLFA) signatures [Red arrows represent significant differences in NLFA signatures of the respective species; black arrows mark non-significant shifts; dotted arrows indicate that statistical analyses were not possible due to low numbers of replicates; differences were tested using DFA for each species]. In brackets after the NLFAs the name of the typical ecological group for which it is a marker is given. NLFA data are untransformed percentages. Length of gradient: 1.5; adjusted explained variation 56.5%; eigenvalues of axis 1 and 2 of 0.44 and 0.33, respectively

The FA composition of the species from the beech forest differed significantly from that of the species from the spruce forest (DFA: Wilks’ λ = 0.52, F_4,19_ = 4.38, p = 0.011). In oribatid mites from the beech forest the relative plant/fungal markers 18:1ω9 and 18:2ω6,9 dominated, with some species, such as *D. riparius*, being more closely associated with 18:2ω6,9, and other species, such as *Steganacarus magnus*, Phthiracaridae and *Tritegeus bisulcatus*, being more closely associated with 18:1ω9 (Fig. 1). By contrast, oribatid mite species from the coniferous forest, such as *Adoristes ovatus*, *Euzetes globulus*, *Liacarus xylariae*, *Nothrus palustris* and *Platynothrus peltifer*, were associated with the bacterial FA markers cy17:0, cy19:0, i15:0, a15:0, 18:1ω7 and 16:1ω7. Overall, the eight studied oribatid mite species occurring in both the beech and spruce forest shifted their FA signatures from fungal/plant FA markers in the beech forest to bacterial markers in the spruce forest; however, this shift was only significant in four species [*Achipteria coleoptrata* (DFA: Wilks’ λ = 0.56, F_4,18_ = 3.58, p = 0.026), *Eupelops hirtus* (Wilks’ λ = 0.31, F_4,12_ = 6.56, p  = 0.0049), *Eupelops plicatus* (Wilks’ λ = 0.082, F_4,5_ = 13.82, p = 0.0065) and *Liacarus xylariae* (Wilks’ λ = 0.0024, F_2,1_ = 207.81, p  = 0.049)]. The shift in *Platynothrus peltifer* and *Damaeus riparius* was not significant (Wilks’ λ = 0.186, F_4,4_ = 4.37, p = 0.091 and Wilks’ λ = 0.514, F_4,8_ = 1.88, p = 0.21, respectively) and the shift in *Nothrus palustris* and *Euzetes globulus* could not be analysed statistically because of the lack of replication.

## Discussion

The aim of this study was to investigate how soil living animals such as oribatid mites are able to cope with changing environmental and/or resource conditions in different forest ecosystems. Therefore, we compared the fatty acid (NLFA) composition of oribatid mite species from a beech forest with that from a spruce forest. The ability to change diet, i.e., trophic plasticity, likely favours coping with changes in environmental conditions. Conversely, the functioning of oribatid mite communities is likely to be maintained if composed of species able to adapt to changing environmental conditions and resource availability. In the present study, trophic plasticity of individual species contributed to the observed shift in channeling energy via fungi, bacteria and plants into oribatid mites in the different forests studied.

### Shifts in trophic niches of oribatid mites

The FA composition of oribatid mites of the studied beech forest differed significantly from that of the studied spruce forest. This indicates that oribatid mites are rather flexible in using different basal resources and suggests that oribatid mites are trophically plastic with this plasticity being similar among oribatid mite species. This plasticity likely contributes to the ability of different species to colonize different forest ecosystems and suggests that they are rather insensitive to changes in the composition of basal resources. This contrasts the response of above ground taxa which are more closely associated with the composition of plant communities and the identity of plant species (Blüthgen et al. 2007; Scherber et al. 2010; Weiner et al. 2010; Blüthgen and Klein 2011).

The FA pattern of oribatid mite species in the studied beech forest indicated intense feeding on fungi and/or plant material (i.e., litter), whereas that in the spruce forest indicated more intensive feeding on bacteria. Less feeding on plant litter in spruce may be due to lower quality of needle litter compared to deciduous litter such as beech (Staaf and Berg 1982; Kaneko and Salamanca 1999; Moore et al. 2006). However, as indicated by rather similar C-to-N ratios in spruce (about 50; Grünzweig et al. 2015) and beech litter (about 40; Mooshammer et al. 2012) diet quality of these litter materials is rather similar. In contrast to the widespread view that fungi dominate in coniferous forests and bacteria dominate in deciduous forests, PLFA analyses in the investigated forests indicated that the fungal energy channel is less pronounced in coniferous than in beech litter, while the bacterial energy channel is more important in coniferous than beech litter (Pollierer et al. 2015). It has been proposed that in general the importance of the bacterial energy channel for the soil animal food web has been underestimated (Pollierer et al. 2012) and this is supported by results of the present study.

Overall, the results of this study and earlier findings (Krause et al. 2019) suggest that soil animals likely are able to respond in a plastic way to changes in the availability of food resources. Despite having a distinct trophic niche and being ascribed to different trophic levels (Maraun et al. 2011) oribatid mites nevertheless have the ability to switch their diet within a certain range.

It is difficult to elicit if the species shifted their diet due to differing resource availability or resource quality. All the studied oribatid mite species have been ascribed either to primary or secondary decomposers (Maraun et al. 2011). None of the taxa shifted these trophic positions completely, i.e., they all remained in the range of primary and secondary decomposers as indicated by stable isotope data. This supports the view that species are flexible within their trophic niche but only rarely switch trophic level.

Results of the present study in part contrast the results of Gan et al. (2014) who used stable isotopes to investigate the ability of oribatid mites to respond to global change processes. They found that oribatid mites exhibit a high degree of trophic specialization and concluded that this limits their ability to cope with changing environmental conditions and food availability. At least in part the different conclusions drawn might be due to different methodology. Most stable isotope data in the study of Gan et al. (2014) were based on pooling a number of individuals. This likely reduced the variance of the stable isotope data and artificially reduced the trophic niche width of oribatid mites. More recent studies using single oribatid mite individuals (Krause et al. 2019) for stable isotope analysis support the view of high trophic plasticity of oribatid mite species.

As stated above, our study also supports Pollierer et al. (2012, 2015) who found that the bacterial energy channel has been underestimated as trophic pathway in soil food webs. Results of the present study suggest that the bacterial energy channel is particularly important if the quality of dead organic material is poor. Spruce litter contains high amounts of lignin, suberin, waxes and other secondary compounds (Kögel-Knabner et al. 1994), and this hampers digestion. Low digestibility of spruce litter likely contributed to the observed switch to a more bacterial-based diet of oribatid mites. In fact, it is known that detritivores increase feeding on litter of low food quality after it has been colonized by microorganisms and plant secondary compounds have been degraded (Wardle et al. 2002).

### Trophic plasticity within oribatid mite species

The trophic shift as indicated by the NLFA pattern between the oribatid mites from beech and from spruce was significant in four species (*Achipteria coleoptrata*, *Eupelops hirtus*, *Eupelops plicatus*, *Liacarus xylariae*). Among the taxa that shifted their diet significantly between beech and spruce forest there was only one primary decomposer (according to stable isotope data and enzyme activities; Maraun et al. 2011; Siepel de Ruiter-Dijkman 1993), i.e., *Achipteria coleoptrata*. This species has limited ability to digest fungi (Gong et al. 2018) and likely mainly feeds on dead plant material. However, in our study it also was associated with bacterial, fungal and plant FA markers indicating that it consumes a mixture of these resources. In the beech forest, it included more plant marker FAs, whereas in the spruce forest it included more bacterial and fungal markers. Presumably, this species fed on litter material colonized little by microorganisms in the beech forest and on needles colonized by bacteria and fungi in the spruce forest.

*Eupelops hirtus*, *Eupelops plicatus* and *Liacarus xylariae* have been assumed to be secondary decomposers, i.e., to mainly feeding on fungi (Maraun et al. 2011; Gong et al. 2018). In the present study, their food resources shifted from plants and fungi in the beech forest to bacteria and fungi in the spruce forest, suggesting that they are indeed secondary decomposers which are able to adapt to locally available resources. Presumably, this contributes to their ability to colonize a wide range of habitats (Fischer and Schatz 2010; Bluhm et al. 2016).

Three species (*Edwardzetes edwardsii*, *Adoristes ovatus*, *Liacarus coracinus*) only occurred in densities sufficient for FA measurement in the spruce forest. *Edwardzetes edwardsii* was more associated with the plant marker 18:1ω9 than the other oribatid mite species from the spruce forest suggesting plant/moss feeding. *Adoristes ovatus* lives inside coniferous needles on the forest floor (Lions and Gourbiere 1988) and was associated with many bacterial FA markers in our study. This indicates that the species is mainly consuming bacteria inside decomposing needles. *Liacarus coracinus* was (compared with the other species from the spruce forest) more associated with the fungal marker 18:2ω6,9 indicating fungal feeding. This is supported by stable isotope data which also grouped this species as fungal feeder/secondary decomposer (Fischer et al. 2010a, b; Bluhm et al. 2015).

FA signatures of five taxa could only be measured in the beech forest (due to low densities or absence in the spruce forests), i.e., *Steganacarus magnus*, Phthiracaridae, *Hermannia gibba*, *Tritegeus bisulcatus* and *Xenillus tegeocranus*. All taxa were closely associated with the plant marker FA 18:1ω9 indicating plant/litter feeding. This is in agreement with stable isotope data which grouped them as primary decomposers (*Steganacarus magnus*, Phthiracaridae; *Hermannia gibba*; Pollierer et al. 2009; Fischer et al. 2010a, b; Maraun et al. 2011; Magilton et al. 2019) or secondary decomposers/fungal feeders (*Tritegeus bisulcatus* and *Xenillus tegeocranus*; Maraun et al. 2011; Bluhm et al. 2015; Corral-Hernandez et al. 2015).

Overall, the results of this study indicate (1) that trophic plasticity in oribatid mites is higher than previously assumed and this may apply to detritivore soil animal species in general, and (2) that oribatid mite species may respond to changing resource conditions in particular by shifting the relative abundance of bacterial- and fungal-based diets. However, the shift in species composition also indicates that some species are unable to adapt to changing resource supply. In sum, the results suggest that trophic plasticity of oribatid mite species, and presumably in soil decomposer animals in general, buffers their response to changes in the availability and quality of resources, and this likely facilitates coping with changes in climate and land use.

## Electronic supplementary material

Below is the link to the electronic supplementary material.

Supplementary file1 (XLSX 31 kb)
